# Regularized quantile regression for SNP marker estimation of pig growth curves

**DOI:** 10.1186/s40104-017-0187-z

**Published:** 2017-07-11

**Authors:** L. M. A. Barroso, M. Nascimento, A. C. C. Nascimento, F. F. Silva, N. V. L. Serão, C. D. Cruz, M. D. V. Resende, F. L. Silva, C. F. Azevedo, P. S. Lopes, S. E. F. Guimarães

**Affiliations:** 1 Department of Statistics, Federal University of Viçosa, Av. P H Rolfs, s/n, University Campus, Viçosa, MG 36570-000 Brazil; 2 Department of Animal Science, Federal University of Viçosa, Av. P H Rolfs, s/n, University Campus, Viçosa, MG 36570-000 Brazil; 3 Department of Animal Science, Iowa State University, Kildee Hall 50011 Ames, Iowa, USA; 4 Department of General Biology, Federal University of Viçosa, Av. P H Rolfs, s/n, University Campus, Viçosa, MG 36570-000 Brazil; 5 Embrapa Forestry, Estrada da Ribeira, km 111, Colombo, PR Brazil; 6 Department of Plant Science, Federal University of Viçosa, Av. P H Rolfs, s/n, University Campus, Viçosa, MG 36570-000 Brazil

**Keywords:** Genome association, Growth curve, Pig, QTL, Regularized quantile regression

## Abstract

**Background:**

Genomic growth curves are generally defined only in terms of population mean; an alternative approach that has not yet been exploited in genomic analyses of growth curves is the Quantile Regression (QR). This methodology allows for the estimation of marker effects at different levels of the variable of interest. We aimed to propose and evaluate a regularized quantile regression for SNP marker effect estimation of pig growth curves, as well as to identify the chromosome regions of the most relevant markers and to estimate the genetic individual weight trajectory over time (genomic growth curve) under different quantiles (levels).

**Results:**

The regularized quantile regression (RQR) enabled the discovery, at different levels of interest (quantiles), of the most relevant markers allowing for the identification of QTL regions. We found the same relevant markers simultaneously affecting different growth curve parameters (mature weight and maturity rate): two (ALGA0096701 and ALGA0029483) for RQR(0.2), one (ALGA0096701) for RQR(0.5), and one (ALGA0003761) for RQR(0.8). Three average genomic growth curves were obtained and the behavior was explained by the curve in quantile 0.2, which differed from the others.

**Conclusions:**

RQR allowed for the construction of genomic growth curves, which is the key to identifying and selecting the most desirable animals for breeding purposes. Furthermore, the proposed model enabled us to find, at different levels of interest (quantiles), the most relevant markers for each trait (growth curve parameter estimates) and their respective chromosomal positions (identification of new QTL regions for growth curves in pigs). These markers can be exploited under the context of marker assisted selection while aiming to change the shape of pig growth curves.

## Background

In general, the study of growth curves is carried out by fitting nonlinear models to weight (dependent variable) and age (independent variable) data. These models are used because they are flexible and have parameters with biological interpretations, such as maturity rate and adult weight.

With the goal of estimating SNP marker effects on parameter estimates of growth curves, Pong-Wong and Hadjipavlou [1] proposed a two-step approach. In the first step, nonlinear models were fitted to the weight-age data of each animal. In the second step, genomic regression models were fitted while considering the parameter estimates from the previous step as the dependent variable. Such an approach allows for the estimation of marker effects based only on the conditional mean of the dependent variable. Specifically, genomic growth curves are defined only in terms of population mean, i.e., the identification of genetically superior individuals in relation to the growth efficiency is based on population mean distribution (quantile 0.5 of a normal distribution of the sampled data).

An alternative approach for the second step that has not yet been exploited in genomic analyses of growth curves is the Quantile Regression (QR) [2]. This methodology allows for the estimation of marker effects at different levels (quantiles) of the variable of interest. Obtaining these effects in specific quantiles allows for a more informative study on the chromosomal regions affecting the growth curve trajectory.

In general, the larger number of markers and the dependence between them due to linkage disequilibrium leads to multicolinearity estimation problems. Thus, methods such as shrinkage estimation, which highlight the high dimensionality and multicollinearity issues, are required. Under a QR framework, this method is named regularized quantile regression (RQR), since the shrinkage (or penalty) parameter regularizes the variance of the markers’ effects, thus performing a direct variable selection framework.

We aimed to propose and evaluate a regularized quantile regression for SNP marker effect estimation of pig growth curves, as well as to identify the chromosome regions of the most relevant markers and to estimate the genetic individual weight trajectory over time (genomic growth curve) under different quantiles (levels).

## Methods

### Animals and genotyping data

Phenotypic data was obtained from the Pig Breeding Farm of the Department of Animal Science of the Federal University of Viçosa, Minas Gerais, and refer to the weights at birth, 21, 42, 63, 77, 105 and 150 days of age. These weights were measured in 345 animals from a F2 outbred population (Brazilian Piau X commercial). More details about this population are found by Azevedo et al. [3] and Band et al. [4].

DNA was extracted at the Animal Biotechnology Lab from Animal Science Department of Federal University of Viçosa. The low-density customized SNPChip with 384 markers was based on the Illumina Porcine SNP60 BeadChip (San Diego, CA, USA, [5]). The number of SNP markers was distributed as follows in the pig chromosomes: (*Sus scrofa*; SSC): SSC1 (*n* = 56), SSC4 (*n* = 54), SSC7 (*n* = 59), SSC8 (*n* = 31), SSC17 (*n* = 25), and SSCX (*n* = 12), totaling 237 SNPs. These markers were selected according to QTL positions that were previously identified in this population by using meta-analyses [6] and fine mapping [7, 8]. Thus, although a small number of markers have been used, the customized SNPchip based on previously identified QTL positions ensures appropriate coverage of the relevant genome regions in this population.

### Statistical analysis

Initially, the logistic nonlinear regression model [9] was fitted to the individual weight-age data:

1 $$ {w}_{ij}=\frac{\alpha_{1 i}}{1+ \exp \left[\left({\alpha}_{2 i}-{t}_j\right)/{\alpha}_{3 i}\right]}+{e}_{ij}, $$

where *w*_*ij*_ is the weight of the animal i at age *t*_*j*_(0, 21, 42, 63, 77, 105 and 150); *α*_1*i*_, *α*_2*i*_ and *α*_3*i*_ are the parameters. If *α*_3*i*_ > 0 then *α*_1*i*_ is the horizontal asymptote as *t*_*j*_ → ∞ (mature weight) and 0 is the horizontal asymptote as*t*_*j*_ → − ∞. If *α*_3*i*_ < 0 these roles are reversed. The parameter *α*_2*i*_ is the *t*_*j*_ value at which the response is *α*_1*i*_/2. It is the inflection point of the curve. The scale parameter *α*_3*i*_ (growth scale) represents the distance on the t-axis between this inflection point and the point where the response is *α*_1*i*_/(1 + *e* ^−1^) ≈ 0.73*α*_1*i*_; *e*_*ij*_ is the independent and normally distributed residual term, ${e}_{ij}\sim N\left(0,{\sigma}_e^2\right).$ In this parameterization, the growth scale parameter is the reciprocal of growth rate on the model presented by Ratkowsky [10].

After obtaining parameter estimates of the logistic model, they were used as dependent variables in a linear model to carry out fixed effect corrections (sex, lot, and halothane gene). The corrected variables were identified based on the residual of the fitted linear model plus the overall mean. Subsequently, the corrected variables (${\widehat{\alpha}}_{1 i}^{\ast },{\widehat{\alpha}}_{2 i}^{\ast }\ \mathrm{and}\ {\widehat{\alpha}}_{3 i}^{\ast }$) were used as dependent variables in a multiple regression model while using SNP markers as the independent variables. This procedure is known in the literature as a two-step approach: in the first step, a growth curve is fitted to the data of each animal, and in the second step, the parameter estimates from the previous step are used as phenotypic values [1, 11].

In the second step, the following genomic model proposed by Meuwissen et al. [12] was fitted separately for each trait (parameter estimates from previous step):

2 $$ {y}_i=\left[\mu +\sum_{k=1}^{237}{x}_{i k}{\beta}_k\right]+{\varepsilon}_i, $$

in which *y*_*i*_ is the corrected phenotype ${\widehat{\alpha}}_{1 i}^{\ast },{\widehat{\alpha}}_{2 i}^{\ast }\ \mathrm{and}\ {\widehat{\alpha}}_{3 i}^{\ast }$ from the first step; *μ*is the general mean; *x*_*ik*_ is the SNP marker, encoded as 2 (AA), 1 (Aa), or 0 (aa); *β*_*k*_ is the effect of the marker k; and *ε*_*i*_ corresponds to the residual term, ${\varepsilon}_i\sim N\left(0,{\sigma}_e^2\right)$.

To obtain the markers’ effects at different levels of the variables (traits defined by ${\widehat{\alpha}}_1^{\ast },{\widehat{\alpha}}_2^{\ast }\ \mathrm{and}\ {\widehat{\alpha}}_3^{\ast }$), the regularized quantile regression [13] was used. This method consists of obtaining the marker effects (*β*_*k*_) that solve the following optimization problem:

$$ {\widehat{\beta}}_s={\mathrm{argmin}}_{\beta}\left\{\sum_{i=1}^{345}{\rho}_{\tau s}\left[{\widehat{\alpha}}_{s i}^{\ast }-\left(\mu +\sum_{k=1}^{237}{x}_{i k}{\beta}_{s k}\right)\right]+{\lambda}_s\sum_{k=1}^{237}\left|{\beta}_{s k}\right|\right\}, $$

where s = 1, 2, and 3 (respectively for each assumed trait, ${\widehat{\alpha}}_1^{\ast }{,\widehat{\alpha}}_2^{\ast }\ \mathrm{and}\kern0.5em {\widehat{\alpha}}_3^{\ast }$); $\sum_{k=1}^{237}\left|{\beta}_{sk0}\right|$ is the sum of the absolute values of the regression coefficients; *λ*_*s*_ is the regularization parameter for each trait; and *τ* ∈ (0, 1) indicates the quantile of interest. This parameter (*λ*_*s*_) is required to avoid multicollinearity problems that are a result of the larger number of highly dependent markers associated with linkage disequilibrium. It leads to the formulation of the RQR.

The parameter *ρ*_*τs*_(.) is denoted as a check function [2] and is defined by:

$$ {\rho}_{\tau s}\left[{\widehat{\alpha}}_{si}^{\ast }-\left(\mu +\sum_{k=1}^{237}{x}_{ik}{\beta}_{sk}\right)\right]=\left\{\begin{array}{l}\tau \cdot \left[{\widehat{\alpha}}_{si}^{\ast }-\left(\mu +\sum_{k=1}^{237}{x}_{ik}{\beta}_{sk}\right)\right],\kern0.75em \mathrm{if}\kern0.5em {\widehat{\alpha}}_{si}^{\ast }-\mu +\sum_{k=1}^{237}{x}_{ik}{\beta}_{sk}>0,\\ {}-\left(1-\tau \right)\cdot \left[{\widehat{\alpha}}_{si}^{\ast }-\left(\mu +\sum_{k=1}^{237}{x}_{ik}{\beta}_{sk}\right)\right],\kern0.5em \mathrm{otherwise}.\end{array}\right. $$

in which *τ* ∈ (0, 1) indicates the quantile of interest. Thus, the values of *β*_*sk*_(*τ*) represent the markers’ effects in the *τ* ^th^ quantile of interest for s^th^ trait.

In this study, for each trait (${\widehat{\alpha}}_1^{\ast }{,\widehat{\alpha}}_2^{\ast }\ \mathrm{and}\kern0.5em {\widehat{\alpha}}_3^{\ast }$), the quantiles *τ* = 0.2 , 0.5 and 0.8 were used to generate results at three distinct levels that may characterize the low, average, and high distribution of the phenotypic values under study (${\widehat{\alpha}}_{1 i}^{\ast },{\widehat{\alpha}}_{2 i}^{\ast }\ \mathrm{and}\ {\widehat{\alpha}}_{3 i}^{\ast }$). Furthermore, these quantiles were chosen to minimize the residual term in previous studies (pilot analysis) by using the same datasets.

In order to verify whether marker effects differ between the quantile levels of the traits (${\widehat{\alpha}}_1^{\ast }{,\widehat{\alpha}}_2^{\ast }\ \mathrm{and}\kern0.5em {\widehat{\alpha}}_3^{\ast }$), the 2.5% most relevant SNPs (highest absolute values) and their *p* values, based on bootstrapped standard error values, were presented. In addition, these SNPs were used to identify possible QTL regions affecting growth traits in pigs.

The Genomic Estimated Breeding Values (GEBV) from RQR were obtained through $GEBV\left(\tau \right)=\widehat{u}=\sum_k{x}_{ik}{\widehat{\beta}}_k\left(\tau \right)$, in which *τ* represents the quantile of interest. Subsequently, the genomic growth curves were obtained for each animal based on GEBV ($\widehat{u}$) according to the following expression:

3 $$ {\widehat{y}}_{ij}=\frac{{\widehat{\mu}}_{{\widehat{\alpha}}_1^{\ast }}+{\widehat{u}}_{{\widehat{\alpha}}_{1 i}^{\ast }}}{\left\{1+ \exp \left[\left({\widehat{\mu}}_{{\widehat{\alpha}}_2^{\ast }}+{\widehat{u}}_{{\widehat{\alpha}}_{2 i}^{\ast }}\right)-\left({\widehat{\mu}}_{{\widehat{\alpha}}_3^{\ast }}+{\widehat{u}}_{{\widehat{\alpha}}_{3 i}^{\ast }}\right){t}_{ij}\right]\right\}},\kern0.75em $$

in which ${\widehat{y}}_{ij}$ is the predicted breeding value for each animal i for the weight at each age (*t*_*ij*_) (j = 0 to 150 d); ${\widehat{\mu}}_{{\widehat{\alpha}}_1^{\ast }}{,\widehat{\mu}}_{{\widehat{\alpha}}_2^{\ast }}$ and ${\widehat{\mu}}_{{\widehat{\alpha}}_3^{\ast }}$ are the means of each trait (parameter estimates for the logistic model); and ${\widehat{u}}_{{\widehat{\alpha}}_{1 i}^{\ast }},{\widehat{u}}_{{\widehat{\alpha}}_{2 i}^{\ast }}$ and ${\widehat{u}}_{{\widehat{\alpha}}_{3 i}^{\ast }}$ are the GEBV of these traits.

Finally, the genetic parameters for the interpretable traits derived from the logistic model (*α*_1_ and *α*_3_) as well as the original traits associated with slaughter weight (SW) and average daily gain (ADG) were estimated by using the following multi-trait model:

4 $$ \left[\begin{array}{c}\hfill {\mathbf{y}}_1\hfill \\ {}\hfill {\mathbf{y}}_2\hfill \end{array}\right]=\left[\begin{array}{cc}\hfill {\mathbf{X}}_{\mathbf{1}}\hfill & \hfill \mathbf{0}\hfill \\ {}\hfill \mathbf{0}\hfill & \hfill {\mathbf{X}}_{\mathbf{2}}\hfill \end{array}\right]\ \left[\begin{array}{c}\hfill {\boldsymbol{\upbeta}}_{\mathbf{1}}\hfill \\ {}\hfill {\boldsymbol{\upbeta}}_2\hfill \end{array}\right]+\left[\begin{array}{cc}\hfill {\mathbf{Z}}_{\mathbf{1}}\hfill & \hfill \mathbf{0}\hfill \\ {}\hfill \mathbf{0}\hfill & \hfill {\mathbf{Z}}_{\mathbf{2}}\hfill \end{array}\right]\ \left[\begin{array}{c}\hfill {\mathbf{g}}_{\mathbf{1}}\hfill \\ {}\hfill {\mathbf{g}}_2\hfill \end{array}\right]+\left[\begin{array}{c}\hfill {\mathbf{e}}_{\mathbf{1}}\hfill \\ {}\hfill {\mathbf{e}}_2\hfill \end{array}\right], $$

where $$ \left[\begin{array}{c}\hfill {\mathbf{y}}_1\hfill \\ {}\hfill {\mathbf{y}}_2\hfill \end{array}\right] $$ is the vector of response variables of traits I and II (*α*_1_ and *α*_3_ with SW and ADG), **X**_**1**_ and **X**_2_ are the fixed-effects design matrix (Sex, Batch, and Halothane presence), **Z**_**1**_ and **Z**_2_ are the random-effects design matrix, and $$ \left[\begin{array}{c}\hfill {\mathbf{e}}_{\mathbf{1}}\hfill \\ {}\hfill {\mathbf{e}}_{\mathbf{2}}\hfill \end{array}\right] $$ is the vector of random residuals of the two traits. It is assumed that $$ \left[\begin{array}{c}\hfill {\mathbf{g}}_{\mathbf{1}}\hfill \\ {}\hfill {\mathbf{g}}_2\hfill \end{array}\right]\sim N\left(\mathbf{0},\mathbf{G}\otimes \mathbf{H}\right) $$, where $$ \mathbf{H}=\left[\begin{array}{cc}\hfill {\sigma}_{g_1}^2\hfill & \hfill {\sigma}_{g_{12}}\hfill \\ {}\hfill {\sigma}_{g_{21}}\hfill & \hfill {\sigma}_{g_2}^2\hfill \end{array}\right] $$ is the additive genetic variance and covariance matrix of the two traits, and $$ \left[\begin{array}{c}\hfill {\mathbf{e}}_{\mathbf{1}}\hfill \\ {}\hfill {\mathbf{e}}_2\hfill \end{array}\right]\sim N\left(\mathbf{0},\mathbf{I}\otimes \mathbf{R}\right) $$, where $$ \mathbf{R}=\left[\begin{array}{cc}\hfill {\sigma}_{e_1}^2\hfill & \hfill {\sigma}_{e_{12}}\hfill \\ {}\hfill {\sigma}_{e_{21}}\hfill & \hfill {\sigma}_{e_2}^2\hfill \end{array}\right] $$ is the residual variance and covariance matrix of the two traits. Finally, **G** is the additive relationship matrix constructed by using 501 pigs and **I** is the identity matrix.

### Computational features

Fitting of the models was carried out by using the *nls* (to fit the logistic nonlinear model in the first step) and *rq* (to fit the regularized quartile regression in the second step) functions of the *stats* and *quantreg* packages [14] of R software [15], respectively. The Mixed Model Analyses were performed in ASReml 3.0 [16].

To obtain the shrinkage parameter values (*λ*), a grid of *λ* values between 0 and 50 was utilized, varying in 0.5 increments. The predictive capacity, defined as the correlation between the estimated and observed values (curve parameters that were obtained from fitting the Logistic model to the weight-age data), was used as a criterion to define the optimal value *λ*.

The computational codes that were implemented in the R software are found on the website of the Statistics Department of the Federal University of Viçosa (2017): https://licaeufv.wordpress.com/scriptrqr_jasb/.

## Results

The summary containing the descriptive statistics of the adjusted phenotypic data is presented in Table 1.

**Table 1:** Means, standard deviations and ranges for weights at seven different ages of F2 outbred population

Age, d	*n*	Mean weight ± SD, kg	Min, kg	Max, kg
0	345	1.20 ± 0.27	0.53	2.13
21	345	4.90 ± 1.00	2.56	8.00
42	345	8.36 ± 1.81	2.66	12.90
63	345	16.29 ± 3.38	7.43	26.53
77	345	21.44 ± 4.39	9.30	34.50
105	345	36.25 ± 6.64	12.79	55.00
150	345	64.97 ± 5.72	39.09	85.20

The summary containing the correlation and descriptive statistics of the adjusted phenotypic data (${\widehat{\alpha}}_{1 i}^{\ast },{\widehat{\alpha}}_{2 i}^{\ast }\ \mathrm{and}\ {\widehat{\alpha}}_{3 i}^{\ast }$) is presented in Table 2.

**Table 2:** Correlation and descriptive statistics among the adjusted phenotypic data (${\widehat{\alpha}}_{1 i}^{\ast },{\widehat{\alpha}}_{2 i}^{\ast }\ \mathrm{and}\ {\widehat{\alpha}}_{3 i}^{\ast }$)

	Correlation			Descriptive statistics		
	${\widehat{\alpha}}_{1 i}^{\ast }$	${\widehat{\alpha}}_{2 i}^{\ast }$	${\widehat{\alpha}}_{3 i}^{\ast }$	Mean ± SD	Min	Max
${\widehat{\alpha}}_{1 i}^{\ast }$	1.00	0.82	0.63	89.43 ± 22.32	35.70	149.85
${\widehat{\alpha}}_{2 i}^{\ast }$	0.82	1.00	0.83	113.18 ± 17.97	72.83	166.43
${\widehat{\alpha}}_{3 i}^{\ast }$	0.63	0.83	1.00	32.03 ± 4.24	22.76	47.29

Considering the aforementioned grid (0 to 50, by 0.5), the shrinkage parameter value that showed the best results in terms of predictive capacity was *λ* = 0.5. Specifically, the predictive capacity ranged between 0.6219 and 0.8252 (Table 3).

**Table 3:** Predictive capacity obtained by means of RQR, considering estimates of the nonlinear regression parameters

Quantile	Trait		
	*α*_1_(*λ* = 0.5)	*α*_2_(*λ* = 0.5)	*α*_3_(*λ* = 0.5)
0.2	0.7143	0.6938	0.6219
0.5	0.8252	0.7889	0.7904
0.8	0.7678	0.7663	0.7636

The mean and standard error for marker effects (${{\widehat{\beta}}_k}^{\prime}\mathrm{s}$) and R^1^ goodness of fit measure for each quantile adjusted model are present in Table 4. The goodness of fit ranged between 0.67 and 0.75 (Table 4).

**Table 4:** Mean, standard error for marker effects and Pseudo R^2^ for each quantile adjusted model

Model	Trait	Mean (Standard error)	Pseudo *R* ^2a^
RQR (0.2)	${\widehat{\alpha}}_1$	0.43(0.37)	0.71
	${\widehat{\alpha}}_2$	0.44(0.45)	0.69
	${\widehat{\alpha}}_3$	0.11(0.14)	0.70
RQR (0.5)	${\widehat{\alpha}}_1$	0.28(0.44)	0.68
	${\widehat{\alpha}}_2$	0.42(0.40)	0.67
	${\widehat{\alpha}}_3$	0.10(0.12)	0.68
RQR (0.8)	${\widehat{\alpha}}_1$	0.48(0.44)	0.75
	${\widehat{\alpha}}_2$	0.52(0.49)	0.74
	${\widehat{\alpha}}_3$	0.13(0.09)	0.75

^a^ Pseudo *R* ^2^ [28]

In order to verify whether the most relevant SNPs for the three approaches (RQR (0.2), RQR (0.5), and RQR (0.8)) were the same, the 2.5% most relevant SNPs for each phenotype (${\widehat{\alpha}}_{1 i}^{\ast },{\widehat{\alpha}}_{2 i}^{\ast }\ \mathrm{and}\ {\widehat{\alpha}}_{3 i}^{\ast }$) were reported (Table 5).

**Table 5:** Absolute values of the estimated effects of the 2.5% most relevant SNP by RQR

Phenotype	Quantile	SNP marker	Estimated effect (abs)	*P*-value^*^	Chromossome (SSC)	Position, cM
	0.20	ALGA0096701	18.93	0.099	17	55.81
	0.20	ALGA0026109	15.29	0.019	4	75.57
	0.20	ALGA0024036	14.98	0.007	4	20.55
	0.20	ALGA0038840	14.50	0.041	7	15.18
	0.20	ALGA0029474	14.15	0.060	4	122.99
	0.20	ALGA0029483	14.07	0.042	4	123.28
	0.50	ALGA0047992	30.89	0.008	8	30.17
Mature	0.50	ALGA0047995	29.47	0.006	8	30.31
Weight,	0.50	ALGA0096701	21.81	0.058	17	55.81
*α*_1_	0.50	ALGA0003761	17.22	0.098	1	50.37
	0.50	ALGA0044299	15.65	0.153	7	110.66
	0.50	ALGA0096707	15.57	0.144	17	55.84
	0.80	ALGA0007216	22.14	0.001	1	160.61
	0.80	ALGA0003761	19.86	0.018	1	50.37
	0.80	ALGA0096701	19.71	0.005	17	55.81
	0.80	ALGA0042986	15.88	0.014	7	90.01
	0.80	ALGA0029474	15.57	0.042	4	122.99
	0.80	ALGA0042863	15.57	0.009	7	86.24
	0.20	ALGA0048131	13.55	0.027	8	35.02
	0.20	ALGA0044519	13.12	0.020	7	115.23
	0.20	ALGA0096701	12.98	0.011	17	55.81
	0.20	ALGA0029483	12.50	0.029	4	123.28
	0.20	ALGA0026109	11.23	0.033	4	75.57
	0.20	ALGA0003761	10.85	0.095	1	50.37
	0.50	ALGA0026100	19.87	0.009	4	75.53
Birth	0.50	ALGA0047995	18.71	0.027	8	30.31
Weight,	0.50	ALGA0048131	18.47	0.029	8	35.02
*α*_2_	0.50	ALGA0047992	16.36	0.062	8	30.17
	0.50	ALGA0039880	14.78	0.047	7	30.13
	0.50	ALGA0021973	14.36	0.015	4	0.28
	0.80	ALGA0048131	17.66	0.007	8	35.02
	0.80	ALGA0005071	17.64	0.002	1	80.44
	0.80	ALGA0042986	16.32	0.005	7	90.01
	0.80	ALGA0029483	15.21	0.010	4	123.28
	0.80	ALGA0003761	15.08	0.025	1	50.37
	0.80	ALGA0026769	14.49	0.073	4	90.18
	0.20	ALGA0049546	3.91	0.015	8	60.04
	0.20	ALGA0029483	3.77	0.005	4	123.28
	0.20	ALGA0096701	3.42	0.011	17	55.81
	0.20	ALGA0021973	3.31	0.014	4	0.28
	0.20	ALGA0048854	3.29	0.031	8	50.17
	0.20	ALGA0048131	3.27	0.035	8	35.02
	0.50	ALGA0048131	5.89	0.004	8	35.02
Growth	0.50	ALGA0021973	4.37	0.023	4	0.28
Rate,	0.50	ALGA0048854	4.13	0.058	8	50.17
*α*_3_	0.50	ALGA0096701	3.66	0.075	17	55.81
	0.50	ALGA0027642	3.62	0.054	4	102.39
	0.50	ALGA0027644	3.36	0.087	4	102.41
	0.80	ALGA0003761	4.43	0.008	1	50.37
	0.80	ALGA0048131	3.74	0.018	8	35.02
	0.80	ALGA0024881	3.61	0.005	4	40.50
	0.80	ALGA0044299	3.34	0.052	7	110.66
	0.80	ALGA0026769	3.07	0.105	4	90.18
	0.80	ALGA0048133	3.01	0.034	8	35.04

^*^ *P*-value calculated using the bootstrap standard error

Table 5 describes the most relevant markers considering the fitting through RQR (0.2). For the mature weight (*α*_1_), the markers are located on chromosomes SSC1, SSC4, SSC7, SSC8, and SSC17 (Table 5). The position of the marker ALGA0096701 on chromosome 17 (55.81 cM) is in accordance with the results of Pierzchala et al. [17], in which the authors found QTL for the slaughter weight at the position 51.1 cM with the cross between Meishan, Pietrain, and European Wild Boar. For birth weight (*α*_2_), the marker ALGA0044519 stands out, which is found in the SSC7 at the position 115.23 cM, next to the QTL for the birth weight found by Guo et al. [18] at the position 120.9 cM for crosses of Large white and Meishan. In terms of growth rate (*α*_3_), the marker that presented with the highest effect is found on chromosome 8. The position of the marker ALGA0049546 at SSC8 (60.04 cM) is close to the position 62.2 cM, as reported by Casas-Carrillo et al. [19] for average daily gain when using families from outbred lines that were selected for high (fast) and low (slow) growth rates.

Considering the RQR (0.5) in Table 5, the most important markers for *α*_1_, *α*_2_ and *α*_3_ are located on chromosomes SSC4 and SSC8 (Table 5; RQR (0.5)). For *α*_1_,, the marker ALGA0047992 stands out, which is found on SSC8 at the position 30.17 cM, which is close to the QTL for slaughter weight found by Beeckmann et al. [20], and at the position 33.9 cM on chromosome 8 in pigs obtained from crosses between Meishan, Pietrain, and European Wild Boar. For the birth weight trait (*α*_2_), the marker with the greatest estimated effect was ALGA0026100. The position of this marker at SSC4 (75.53 cM) is close to the position at 74.4 cM reported by Walling et al. [21] for body weight at birth. For *α*_3_, the position of marker ALGA0048131 on SSC8 (35.02 cM) was close to the position 33.1 cM reported by Beeckmann et al. [20] who used data from an experimental cross between Meishan, Pietrain, and European Wild Boar for average daily gain (Table 5; RQR (0.5)).

Considering the RQR (0.8) in Table 5, the most significant SNPs for *α*_1_, *α*_2_ and *α*_3_ are located on chromosomes SSC1 and SSC8 (Table 5; RQR (0.8)). Regarding the mature weight trait (*α*_1_), the marker with the highest absolute value pertaining to the estimates of the parameter effect is ALGA0007216. This marker is located on chromosome 1 (160.61 cM). Chen et al. [22] used a pig population comprised of Yorkshires and Meishans to find significant QTLs for slaughter weight at the position 122.4 cM of SSC1, i.e., close to the position 160.61 cM of the ALGA0007216 marker (Table 5; RQR (0.8)).

Another interesting result that was observed through RQR is the simultaneous existence of important markers for different traits (Table 5). This fact is important for breeding, since pleiotropy is the main factor in genetic correlation. Specifically, for RQR (0.5) (Table 5), two markers (ALGA0047992 and ALGA0047995) were simultaneously important for the mature weight (*α*_1_) and birth weight (*α*_2_) traits. In addition, three SNPs for RQR (0.2) (ALGA0096701, ALGA0026109, and ALGA0029483) and one for RQR (0.8) (ALGA0042986) were simultaneously relevant for *α*_1_ and *α*_2_.

Considering the traits *α*_1_ (mature weight) and *α*_3_ (growth rate), two (ALGA0096701 and ALGA0029483), one (ALGA0096701), and one (ALGA0003761) markers were simultaneously important for the methodologies RQR (0.2), RQR (0.5), and RQR (0.8), respectively. For the traits *α*_2_ (birth weight) and *α*_3_ (growth rate), three markers in the RQR (0.2) methodology (ALGA0048131, ALGA0096701, and ALGA0029483), two in the RQR (0.5) methodology (ALGA0048131 and ALGA0021973), and three in the RQR (0.8) methodology (ALGA0003761, ALGA0026769, and ALGA0048131) were simultaneously relevant for these two traits (Table 5).

**Fig. 1:**
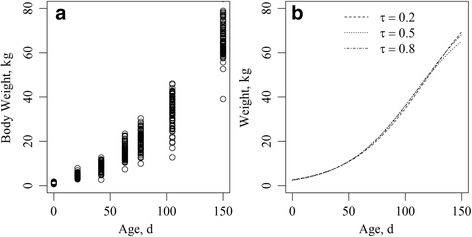
**a** Body weight (BW) data of animals over time. Each dot in the figures represents the BW of an animal, and **b** Genomic growth curves for each Regularized Quantile Regression (RQR), for quantiles 0.2, 0.5, and 0.8

The three genomic growth curves (*τ* = 0.2 , 0.5 , 0.8) that were obtained based on all of the data are shown in Fig. 1b. The estimated curve based on the three quantiles showed a similar pattern until 100 d. After that, differences in the estimated growth curves increased with time (Fig. 1b). This result was expected given the increase in the heterogeneity of variances that were presented at the final evaluated times, 100 and 150 d (Fig. 1a).

**Fig. 2:**
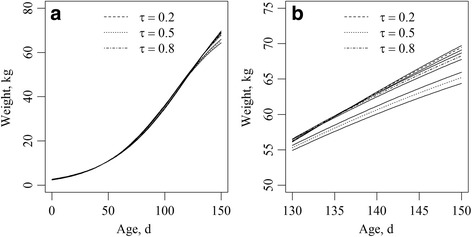
**a** Genomic growth curves for each Regularized Quantile Regression (RQR), for quantiles 0.2, 0.5, and 0.8 and their confidence intervals. **b** Genomic growth curves for each RQR, for quantiles 0.2, 0.5, and 0.8 and their confidence intervals. The genomic growth curves are highlighted within the distribution ranges of Age (130 to 150 d) and Weight (50 to 75 kg)

The genomic growth curves for each RQR, for quantiles 0.2, 0.5, and 0.8 and their confidence intervals showed significant differences (based on non-overlapping confidence intervals) only in terms of mature weight (Fig. 2a). These differences are highlighted in Fig. 2b.

Estimates of genetic parameters (heritability, and genetic and phenotypic correlations) are presented in Table 6. Estimates of heritability for growth curve parameters were moderate, with 0.447 ± 0.200 and 0.4991 ± 0.164, for parameters *α*_1_ and *α*_3_, respectively. The original traits (SW and ADG) had low heritability estimates, with 0.214 ± 0.127 and 0.094 ± 0.087, for SW and ADG, respectively.

**Table 6:** Genetic parameters^a^ (standard error) for growth curve parameters, ADG, and SW

Traits^b^	*α*_1_	*α*_3_	ADG	SW
*a*_1_	0.447 (0.200)	0.809 (0.191)	−0.613 (0.390)	0.404 (0.113)
*a*_3_	0.759 (0.030)	0.491 (0.164)	−0.681 (0.229)	-
ADG	0.047 (0.090)	−0.451 (0.06)	0.214 (0.127)	0.892 (0.677)
SW	0.662 (0.051)	−0.191 (0.080)	0.687 (0.039)	0.094 (0.087)

^a^Heritability, and genetic and phenotypic correlations presented on the diagonal, lower off-diagonal, and upper off-diagonal, respectively

^b^ *α*_1_ asymptotic weight (mature body weight), *α*_3_ inflection point, *ADG* average daily gain, *SW* slaughter weight

Estimates of genetic and phenotypic correlations are presented in the off-diagonals (Table 6). Between the interpretable growth curve parameters (*α*_1_ and *α*_3_) with the original correspondent traits (SW and ADG), correlations were, respectively, highly positive and negative, with a positive genetic correlation estimated for parameters *α*_1_ and SW (0.404 ± 0.113) and a negative genetic correlation estimated for *α*_3_ with ADG (−0.681 ± 0.229). Phenotypic correlations between interpretable growth curve parameters with slaughter weight (SW) and average daily gain (ADG) traits were also moderately positive and negative, with 0.662 ± 0.051 for *α*_1_ with SW and −0.451 ± 0.06 for *α*_3_ with ADG.

## Discussion

In this study, we aimed to propose and evaluate a regularized quantile regression (RQR) for SNP marker effect estimation on pig growth curves and to estimate the genetic weight trajectory over time (genomic growth curve) under different quantiles (levels). In order to do so, a real data set consisting of 345 animals from an F2 outbred population with information on 237 SNP markers, randomly distributed over six chromosomes, was used. The phenotypic data refers to the weight at birth, 21, 42, 63, 77, 105, and 150 days of age. To estimate SNP marker effects for growth curves, we used a two-step approach [1]. In the first step, we fitted logistic nonlinear models to the data of each animal, and in the second step, genomic regression models were fitted while considering the estimated parameters from the previous step as the phenotypic values. We obtained the three genomic growth curves for the three evaluated quantiles (*τ* = 0.2 , 0.5 , 0.8). Finally, the genetic parameters for the interpretable traits of the logistic model (*α*_1_ and *α*_3_) and the original traits, slaughter weight and average daily gain, were estimated.

Quantile regression (QR) can be used to provide a more complete statistical analysis of the stochastic relationships among random variables. In general, the chosen quantiles depend entirely on the purpose of the study, i.e., we can study all distributions or only some parts by defining specific quantiles. In this study, with the aim of representing three distinct levels that characterize low, average, and high distributions of the phenotypic values (estimated parameters while considering a logistic nonlinear model), we choose, *τ* = 0.2 , 0.5 , 0.8.

The use of RQR to estimate SNP marker effects and obtain the estimated genomic growth curve was efficient since it was possible to construct genomic growth curves and find the most relevant markers, which thus allows for the identification of QTL regions at different levels of interest. Besides that, R^1^ goodness of fit measures ranging from 0.67 to 0.75 indicating that the model fits well for the observations.

Unlike traditional methods that are based on conditional expectations, E(Y|X), RQR allows us to fit regression models on different parts of the distribution of the variable response, therefore enabling a more complete understanding of the phenomenon under study [2, 23]. Besides, the heterogeneous variance over time (Fig. 1a) indicates that there is not a single rate of change that characterizes changes in the probability distribution, therefore indicating that RQR is a good tool to deal with those situations. Also, the predictive capacity that was obtained by means of RQR (Table 3) was better than that obtained by Silva et al. [24].

The advantages of RQR, such as studying different parts of the distribution of the variable response, can be combined with those from the two-step approach. Specifically, the two-step approach enables us to obtain the genomic values for each observed time (t_j_), as well as to estimate the weight for any other time of interest within the measured range before this weight is attained [24].

Based on the results, it is possible to note that RQR allows for the identification of markers close to QTLs at different distribution levels of the phenotypic values of interest. The regions indicated by RQR coincide with the results of several studies in which the authors found QTL for the traits that were evaluated in this study.

The use of quantile regression to estimate genomic curves based on three contrasting quantiles in our population was efficient when it came to producing distinct growth curves. Specifically, we can see in Fig. 2b that the final BW of the genomic growth curves was statistically different; in other words, the growth behavior over time changed in terms of mature weight. In fact, this result shows that RQR is a statistical method that could be effectively used to estimate more than a single mean behavior, thereby providing a more complete picture of the relationships between variables.

The genetic correlations between *α*_1_ and *α*_3_ with BW and ADG had, respectively, a high positive and negative genetic correlation, which indicates that *α*_1_ and *α*_3_ have the potential to be used as selection tools to improve SW and ADG. Additionally, the high genetic correlation between *α*_1_ with *α*_3_ and SW with ADG enable us to understand causes of SNPs’ pleiotropic effects. These results are in agreement with Silva et al. [24], who found significant genetic correlation between the interpretable traits of logistic model (${r}_{\alpha_1,{\alpha}_3}=-0.69$) in the same populations that were used in this study. The difference between the signals of genetic correlation estimates observed in the present study is due to the different Logistic model parameterizations. Specifically, our approach uses the parameterization presented in Pinheiro and Bates [9], where the growth scale parameter (*α*_3_) is the reciprocal of growth rate [10, 24].

The study of different distribution levels of the variable of interest using QR has been successfully performed in medicine by Beyerlein et al. [25], who used QR in GWAS (Genome-Wide Association Study) analysis in human genetics where they emphasized statistical and biological advantages when estimating marker effects in different quantiles of the phenotypic distribution. Sun et al. [26] proposed to use QR to identify hypermethylated CpG islands (CGIs) that can be associated with breast and ovarian cancer. They concluded that the quantile level between 80 and 90% is the best strategy to identify methylated and unmethylated CGIs. Moreover, regularized quantile regression has already been successfully evaluated for analyzing ultra-high dimension data [27]. These authors demonstrated that QR greatly enhances existing tools for large dimensional data analysis, since it revealed a substantial reduction in model complexity when compared with alternative methods.

However, even though the use of RQR is promising and efficient, more studies are needed to address the choice of the shrinkage parameter value, which is always critical to find as it can be defined by using a grid of values, cross-validation, or by using a Bayesian approach. Another issue about the use of RQR is the choice of the quantile. There are a lot of quantiles that can be used; therefore, finding the best one to explain the functional relationship is a challenge.

## Conclusions

The proposed model enabled the discovery, at different levels of interest (quantiles), of the most relevant markers for each trait (growth curve parameter estimates) and their respective chromosomal positions (identification of new QTL regions for growth curves in pigs). Furthermore, RQR enabled the construction of genomic growth curves, which identified genetically superior individuals in relation to growth efficiency.

## Abbreviations

ADG: Average daily gain
BW: Body weight
GEBV: Genomic estimated breeding value
GWAS: Genome-Wide Association Study
QR: Quantile Regression
QTL: Quantitative trait loci
RQR: Regularized Quantile Regression
SNP: Single-nucleotide polymorphism
SSC: *Sus scrofa*
SW: Slaughter weight
